# Effect of Acute Exercise on Prefrontal Oxygenation and Inhibitory Control Among Male Children With Autism Spectrum Disorder: An Exploratory Study

**DOI:** 10.3389/fnbeh.2020.00084

**Published:** 2020-06-04

**Authors:** Emily Bremer, Jeffrey D. Graham, Jennifer J. Heisz, John Cairney

**Affiliations:** ^1^Faculty of Kinesiology and Physical Education, University of Toronto, Toronto, ON, Canada; ^2^Department of Family Medicine, McMaster University, Hamilton, ON, Canada; ^3^Department of Kinesiology, McMaster University, Hamilton, ON, Canada; ^4^School of Human Movement and Nutrition Sciences, University of Queensland, St Lucia, QLD, Australia

**Keywords:** physical activity, executive function, autism spectrum disorder, psychophysiology, fNIRS

## Abstract

**Objective**: Children with autism spectrum disorder (ASD) experience significant challenges in executive functioning. Emerging evidence suggests exercise may improve executive functioning among children; however, these effects and their mechanisms have not been fully explored among children with ASD. The purpose of this study was to explore the acute effect of exercise on cerebral oxygenation within the prefrontal cortex and inhibitory control among male children with ASD.

**Method**: Participants (*N* = 12) were 8–12 years of age with a diagnosis of ASD. A within-subject crossover design was employed. Participants completed three 20-min conditions on separate days: circuit-based workout, treadmill walking, and sedentary control. Pre- and post- each condition participants completed a cancellation task (Leiter-3) as a measure of inhibitory control and cerebral oxygenation was concurrently assessed using functional near-infrared spectroscopy (fNIRS). Heart rate, affect, perceived exertion, motivation, and self-efficacy were measured throughout the experiment as manipulation checks and potential psychological mechanisms. A series of repeated measures ANOVAs were conducted to examine intervention effects.

**Results**: Results demonstrated medium-to-large interaction effects (time by condition) for cerebral oxygenation ($\eta_{\text{p}}^{2}$ = 0.237) and inhibitory control ($\eta_{\text{p}}^{2}$ = 0.118). *Post hoc* analyses revealed that the circuit exercise condition elicited the largest changes in both outcomes. The manipulation checks indicated that the exercises were completed as intended.

**Conclusion**: These findings suggest that exercise may be a feasible intervention for enhancing executive functioning in children with ASD. More research with larger samples is needed to replicate these findings.

## Introduction

Autism spectrum disorder (ASD) is a neurodevelopmental disorder that results in challenges in the areas of social communication and social interactions, along with the presence of restricted or repetitive behaviors (American Psychiatric Association, 2013). While the etiology of ASD is complex and includes both genetic and environmental factors (Currenti, 2010), much work has also focused on the widespread disrupted neural connectivity that is present in ASD (Belmonte, 2004; Schipul et al., 2011; Wass, 2011). Brain imaging research, for example, points to connectivity issues between the frontal cortex and other brain regions, which is thought to contribute to the behavioral characteristics of ASD (Wass, 2011; Vissers et al., 2012). Further, the prefrontal cortex, which continues to develop through childhood, is thought to be particularly impacted by these connectivity issues (Just et al., 2007).

Parallel to the core features of ASD, and in line with the widespread neural disruptions, previous research has also demonstrated significant impairments in executive functioning among this population (Demetriou et al., 2018). Executive functions are higher-order cognitive processes, originating in the prefrontal cortex, which enables us to control and direct our behavior (Diamond, 2013). Executive functions include three core processes including inhibition (i.e., self-control or interference control), working memory, and cognitive flexibility (i.e., set-shifting or mental flexibility; Miyake et al., 2000; Lehto et al., 2003). These skills are essential for virtually all aspects of life including physical and mental health, academic and vocational success, and social development (Diamond, 2013). Thus, impairments in executive functioning can have a debilitating impact across multiple aspects of functioning.

Interventions for children with ASD typically focus on addressing the behavioral challenges associated with the disability through, for example, intensive behavioral interventions. However, few interventions have directly addressed the deficits in executive functioning, rather than the behaviors associated with these deficits. One example of a successful intervention targeting executive functions for children with ASD employed cognitive behavioral strategies in a school-based program and found significant gains in the areas of problem-solving, flexibility, and planning (Kenworthy et al., 2014). Yet, the potential mechanisms behind these changes remain unknown.

Exercise is a form of intervention that has been gaining traction as a way to improve aspects of executive functioning in children. While the results of recent reviews are mixed (Hillman and Biggan, 2017; Diamond and Ling, 2018; Gunnell et al., 2019), the guiding principle is that both acute and chronic exercise results in several physiological and psychological changes that, in turn, elicit improvements in brain-based processes (Lambourne and Tomporowski, 2010; Stimpson et al., 2018). While traditional forms of aerobic exercise (e.g., running) have been the focus of much of this research (Gunnell et al., 2019; Pontifex et al., 2019), the effects of exercise on executive functions may not be equal across exercise types. For example, previous research has suggested that cognitively engaging activities (i.e., physical activity paired with cognitive/mental effort) may elicit greater improvements in executive functioning than aerobic exercise alone (Best, 2010; Schmidt et al., 2015; Diamond and Ling, 2018). Further, resistance training has received a lot less attention in the literature but has demonstrated positive results on measures of inhibition (Soga et al., 2018). Several potential mechanisms are driving the effects of exercise on cognition. For example, increased arousal leads to a cascade of factors including activation of the reticular activating system and the release of catecholamines and neurotrophic factors (Voss et al., 2013; McMorris, 2016; Pontifex et al., 2019). Further, exercise may increase cerebral blood flow regulation, resulting in improved cognitive processing through an increase in resource availability (i.e., availability of oxygenated hemoglobin) and waste clearing (i.e., removal of deoxygenated hemoglobin; Ogoh and Ainslie, 2009). Numerous intermediary psychological mechanisms may be driving these changes. For example, exercise can lead to improved affect (Ekkekakis et al., 2011), which may then impact performance on later cognitive tasks. Further, self-efficacy to perform cognitive tasks may also be improved through changes in both affective and physiological states following exercise (Bandura, 1997).

Despite the challenges in executive functioning experienced by children with ASD, and the potential for exercise to reduce these challenges, little work in this area has focused specifically on this population. There is evidence of behavioral improvements in the areas of social-emotional functioning and a reduction in repetitive or stereotyped behaviors following exercise in children with ASD (Bremer et al., 2016; Ferreira et al., 2019; Huang et al., 2020; Tse, 2020), with these improvements hypothesized to be related to improvements in executive functioning (Bremer et al., 2016). Further, a recent meta-analysis of the effect of exercise interventions on cognition reported a large effect for improvements in specific aspects of cognition (e.g., time on task) for children with ASD (Tan et al., 2016). Our interpretation of this effect is, however, limited as it was derived from only six studies and only one of these studies (Anderson-Hanley et al., 2011) included measures of executive functioning as an outcome. Since this review, two additional studies have shown positive changes in executive functions in children with ASD following 12-week table tennis and basketball interventions, respectively (Pan et al., 2017; Tse et al., 2019). Additionally, Memari et al. (2017) found a significant positive correlation between cognitive flexibility and physical activity in children with ASD; although, the cross-sectional design of the study limits our understanding of causality in this relationship. Taken together, these results suggest there is potential for exercise to improve executive functions in children with ASD. The current literature, however, has failed to test potential intermediary variables that may account for changes in executive functions, as well as the differential effects of varying types of exercise on executive functions in this population.

This exploratory study examined the effect of two different types of acute exercise (treadmill walking and a circuit-based interval workout), in comparison to a sedentary control condition, on changes in cerebral oxygenation within the prefrontal cortex and inhibitory control in male children with ASD. We hypothesized that both exercise conditions would result in greater improvements to both variables than the sedentary control condition. As a secondary research question, we also explored changes in potential intermediary psychological (self-efficacy, motivation, affect, and perceived exertion) and physiological (heart rate) variables.

## Materials and Methods

### Participants and Design

**Figure 1:**
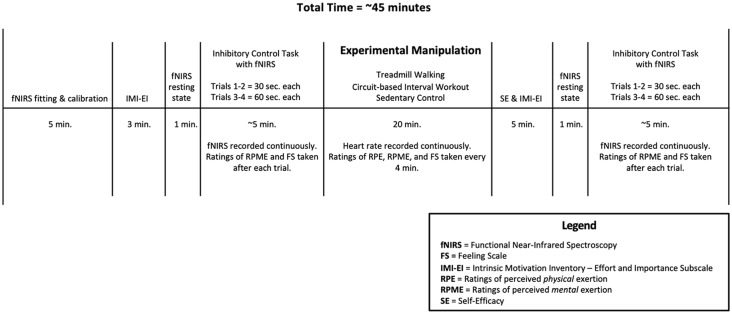
Study protocol.

Participants were 12 males with ASD between 8–12 years of age (M_age_ = 11.1, SD = 1.3 years). Eligibility criteria included no co-occurring physical health conditions (e.g., unstable heart condition) that would preclude participants from safely engaging in physical activity and the ability to follow dual-step instructions, as reported by the participants’ parent. The study utilized a within-subject randomized experimental design with one independent variable (condition, consisting of three levels—treadmill walking, circuit-based interval workout, and sedentary control) and two primary dependent measures (change in cerebral blood flow and inhibitory control, respectively). The study was approved by the Hamilton Integrated Research Ethics Board, parents provided informed written consent, and participants provided informed written assent before participation in the study. Figure 1 provides an overview of the experimental design.

### Experimental Manipulation

Participants completed three experimental conditions, in a random order, separated by a minimum of 48 h. Randomization was determined using a random number generator (randomizer.org) before study entry. The manipulations included two exercise conditions (steady-state and interval) and one sedentary control condition. Each condition was 20 min in length. Heart rate was recorded continuously during each condition using a Polar V800 heart rate monitor and chest strap, with a target heart rate for the exercise conditions of 120–160 beats per min (bpm; approximately 60–80% of predicted maximal heart rate, Gelbart et al., 2017).

#### Treadmill Walking

Treadmill walking was chosen as a steady-state, aerobic exercise as this type of exercise is commonly employed in the acute exercise-cognition literature (Pontifex et al., 2019). The condition consisted of walking on a treadmill with the speed and incline starting at 1.7 mph and 10%, respectively, to meet target heart rate zones. The speed and incline were adjusted, if needed, to maintain the target heart rate.

#### Circuit-Based Interval Workout

The circuit-based interval exercise condition included five exercises, completed in a circuit, for a total of three sets. This condition was chosen to represent a more ecologically valid type of workout and included exercises that could be completed at home or in school or clinical settings. The exercises included jumping jacks, medicine ball (2 lb) chest press, squat jumps, a seated row with a light resistance band, and alternating step-ups on an exercise step. Each exercise was completed for 45 s, followed by a 20-s rest and transition to the next exercise. A 2-min rest was provided between each set. The timing of the exercises and transitions between activities were guided through a commercially available iPad application (Seconds Pro Interval Timer, Runloop Limited). Participants were verbally encouraged to maintain a steady pace throughout the exercises to stay in the target heart rate zone.

#### Sedentary Control

The sedentary control condition consisted of watching an age-appropriate children’s movie while seated in a chair in the exercise room. Considered a cognitive engagement control, this is the most commonly used control condition in the acute exercise cognition literature (Pontifex et al., 2019). Further, this was considered a typical activity in which a child with ASD may engage in their free time (Jones et al., 2017).

### Primary Outcome Measures

#### Cerebral Oxygenation

**Figure 2:**
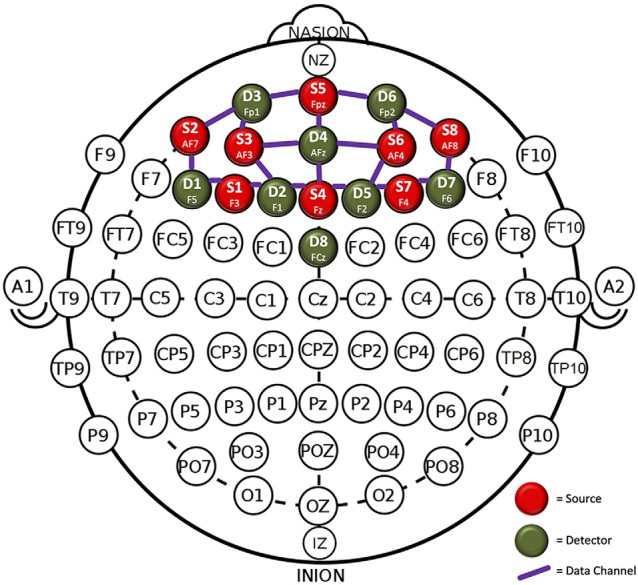
Optode placement in the prefrontal cortex.

A 20-channel, continuous wave, functional near-infrared spectroscopy (fNIRS) system (NIRSport, NIRx Medical Technologies) was used to measure the relative change in oxygenated hemoglobin (oxy-Hb) within the prefrontal cortex during the inhibitory control task. Previous research has demonstrated a high level of agreement between oxy-Hb and blood oxygen level-dependent (BOLD) signals from fMRI, which is considered the gold standard for measuring cerebral blood flow regulation (Strangman et al., 2002). The system is comprised of eight sources and eight detectors, resulting in 20 channels with a sampling frequency of 7.81 Hz. The optodes were placed in the cap based on the 10-10 system (Chatrian et al., 1985) at a uniform distance of 3 cm apart to acquire the hemodynamic measures from the prefrontal cortex (Figure 2). After placing the optodes in the cap, a thick black over-cap was used to cover the optodes to avoid interference from environmental light.

The raw fNIRS data were recorded continuously during the inhibitory control task using the NIRStar acquisition software. The raw data was then processed offline with nirsLAB. First, the task onsets and rest periods were defined. Signal-to-noise performance was evaluated using variation coefficients (CV), which is the standard deviation divided by the mean from all of the raw data in the measurement time series, by channel, and expressed as a percentage (Piper et al., 2014; Kenville et al., 2017). In line with previous literature, channels were removed when the CV exceeded 15% (Piper et al., 2014; Kenville et al., 2017). A digital band-pass filter with a low cut-off frequency of 0.0033 Hz and a high cut-off frequency of 0.02 Hz was applied to the raw data to remove high-frequency noise and cardiovascular artifacts. Hemodynamic states were then computed and the data were exported to Microsoft Excel for post-processing analyses. The mean oxy-Hb across all channels was calculated for each of the four blocks of the inhibitory control task. Change from pre- to post-test in the average oxy-Hb (in μ mol) was considered the primary outcome from this variable.

#### Inhibitory Control

Inhibitory control was assessed with the Attention Sustained subtest of the Leiter International Performance Scale, 3rd Edition (Roid et al., 2013). The Attention Sustained subtest measures inhibitory control through a cancellation task that requires the participant to cross out as many target items on a page as they can in a given time. The subtest includes four trials, with the first two trials lasting 30 s and the second two trials lasting 60 s. Each of the four trials is completed on a separate page and has a different target item for the participant to find. Each page is full of items similar to the target and the participant needs to cross out only the target items while inhibiting the desire to cross out similar, non-target, items. The four trials progress in difficulty by including previous target items in subsequent trials, increasing the complexity of the target items, and moving from a linear to the random arrangement of the items on the page. Before each of the four trials, participants are given a practice trial on a miniature version of the task to ensure task understanding. Scores are calculated as the total number of target items correctly crossed out, minus the total number of commission errors. The total number of target items for each of the four trials is 32, 64, 69, and 52, respectively, with the highest total score (sum of trials 1–4) being 217 (Roid et al., 2013). The subtest was completed twice (pre- and post-experimental manipulation) per study appointment. The change in total score from pre- to post-test was considered the primary outcome from this variable.

### Secondary Outcome Measures and Manipulation Checks

#### Affect

The Feeling Scale (Hardy and Rejeski, 1989) was used to measure affective states at baseline, following each of the four inhibitory control trials and at 4-min intervals throughout the experimental manipulation. The Feeling Scale is an 11-point bipolar single-item scale that ranges from −5 (*very bad*) to +5 (*very good*) along a displeasure-pleasure continuum. The average of the four affect ratings following the inhibitory control trials was considered a secondary outcome, while the average of the five ratings during the experimental manipulation was considered a manipulation check.

#### Ratings of Perceived Mental Exertion

Participants rated their perceived mental exertion (RPME) using an adapted version of Borg’s CR-10 scale (Borg, 1998) following each of the four inhibitory control trials and at 4-min intervals throughout the experimental manipulation. Participants were instructed to rate their perceived mental exertion from 0 (no exertion at all) to 12 (absolute maximum), with 10 (extremely strong) representing the highest mental exertion they had ever experienced. The average of the four RPME ratings following the inhibitory control trials was considered a secondary outcome, while the average of the five ratings during the experimental manipulation was used as a manipulation check.

#### Ratings of Perceived Physical Exertion

Participants rated their perceived physical exertion (RPE) using Borg’s CR-10 scale (Borg, 1998), at 4-min intervals throughout the experimental manipulation to determine the extent to which they were physically exerting themselves during each of the three conditions. Participants were instructed to rate their perceived physical exertion from 0 (no exertion at all) to 12 (absolute maximum), with 10 (extremely strong) representing the highest physical exertion they had ever experienced. The average of the five ratings during the experimental manipulation was used as a manipulation check.

#### Intrinsic Motivation

The motivation for performing the inhibitory control task was assessed at pre- and post-test using the effort and importance subscale from the Intrinsic Motivation Inventory (Ryan, 1982). The subscale consists of five items that are rated on a Likert scale ranging from 1 (*not at all true*) to 7 (*very true*). A total motivation score was calculated as the mean of the five items and change from pre- to post-test was considered a secondary outcome.

#### Task Self-efficacy

Self-efficacy to perform the inhibitory control task was assessed once (i.e., before performing the post-test) per appointment, using a four-item, 11-point, scale adhering to recommendations by Bandura (1997, 2006) for measuring self-efficacy. The scores from each of these four items were averaged to create a task self-efficacy score ranging from 0 to 10, which was considered a secondary outcome.

#### Heart Rate

Heart rate was measured continuously during the experimental conditions using a Polar H1 chest strap synced to a Polar V800 watch (Polar Canada, Lachine, Quebec). The average heart rate, in beats per minute, over the 20-min condition was used as a manipulation check of exercise intensity.

### Sample Descriptive Characteristics

#### ASD Severity

Parents completed the Gilliam Autism Rating Scale—Third Edition (GARS-3; Gilliam, 2014). The GARS-3 yields standard scores, percentile ranks, severity level, and the probability of Autism and has been shown to accurately discriminate children with ASD from those without (sensitivity = 0.97 specificity = 0.97; Gilliam, 2014).

#### Trait Executive Functioning

Parents completed the Behavior Rating Inventory of Executive Functions (BRIEF; Gioia et al., 2000). The instrument assessed the following domains of executive functioning: inhibition, shifting, emotional control, working memory, and planning/organizing. Scores were totaled to give a Global Executive Composite. Parent test-retest reliability scores from the normative sample of 1,419 parents of children aged 5–18 are 0.82 (Gioia et al., 2000).

#### Body Composition

Height was measured to the nearest 0.1 cm using a Seca SC264 stadiometer (Chino, California) and weight was measured to the nearest 0.1 kg using a Seca SC869 digital scale (Chino, California). Body mass index (BMI) was calculated and participants’ weight status was classified using the IOTF cut-points (Cole and Lobstein, 2012).

### Statistical Analyses

All statistical analyses were conducted using SPSS 25 (IBM Corp., 2017). Descriptive statistics were computed for all study variables. Separate two-way repeated-measures ANOVAs were computed to assess differences in cerebral oxygenation, inhibitory control, affect, RPME, and motivation from pre- to post-test by the condition. Separate one-way ANOVAs were used to assess differences in means between conditions for self-efficacy and the manipulation checks. Planned *post hoc* comparisons using paired *t*-tests were conducted on the primary outcomes. Effect sizes for the repeated measures ANOVAs are reported as partial eta squared ($\eta_{\text{p}}^{2}$) and the values for small, medium, and large effects are 0.01, 0.06, and 0.14, respectively (Cohen, 1992). Effect sizes for the *post hoc* analyses are reported as Cohen’s *d_rm_* and the values for small, medium, and large effects are 0.2, 0.5, and 0.8, respectively (Cohen, 1992).

#### Sensitivity Analysis

Due to challenges with recruitment, our sample size was limited to 12 participants. Therefore, a *post hoc* sensitivity analysis was conducted to determine the size of the effect needed to reach statistical significance (G*Power version 3.1.9.2; Faul et al., 2009). Based on our sample size of *N* = 12, with power = 0.80, and alpha = 0.05, a large effect of $\eta_{\text{p}}^{2}$ = 0.219 was necessary to achieve statistical significance.

## Results

The demographic characteristics of the participants are presented in Table 1. The majority of participants (75%) had an autism severity rating of level 2 (i.e., requiring substantial support), while one participant was considered level 1 (i.e., requiring support), and two participants were considered level 3 (i.e., requiring very substantial support), respectively.

**Table 1:** Demographic characteristics of the participants.

Variable	Mean (SD)
Age	11.1 (1.3)
BMI	18.2 (2.5)
GARS autism index	90.0 (17.0)
BRIEF global executive composite	71.1 (9.4)

*Note: BMI, body mass index; GARS, Gilliam Autism Rating Scale; BRIEF, Behavior Rating Inventory of Executive Function*.

### Primary Analyses

**Figure 3:**
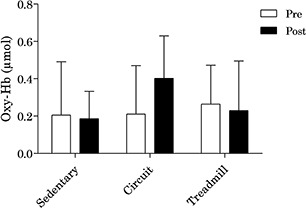
Change in oxygenated hemoglobin in the prefrontal cortex by condition.

In regard to changes in cerebral oxygenation, there was a small effect size for the main effect of time (*F*_(1,7)_ = 0.3, *p* = 0.597, $\eta_{\text{p}}^{2}$ = 0.042) and a medium effect size for the main effect of condition (*F*_(2,14)_ = 0.8, *p* = 0.469, $\eta_{\text{p}}^{2}$ = 0.103). A large effect size was present for the interaction effect (*F*_(2,14)_ = 2.2, *p* = 0.151, $\eta_{\text{p}}^{2}$ = 0.237). *Post hoc* analyses indicated that there was a large effect on cerebral oxygenation following the circuit condition [*M*_(SD)_ = 0.217 (0.315); *t*_(7)_ = 1.95, *p* = 0.092, *d*_rm_ = 0.85], but not following the treadmill [*M*_(SD)_ = 0.035 (0.319); *t*_(7)_ = 0.306, *p* = 0.769, *d*_rm_ = 0.14], and sedentary conditions [*M*_(SD)_ = 0.081 (0.279); *t*_(7)_ = 0.823, *p* = 0.438, *d*_rm_ = 0.39]. Change in cerebral oxygenation by condition is presented in Figure 3.

**Figure 4:**
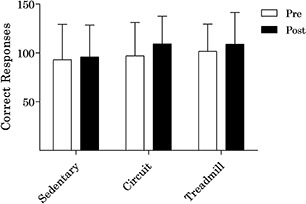
Change in inhibitory control by condition.

In regard to inhibitory control, there were large effect sizes for the main effects of time (*F*_(1,11)_ = 13.2, *p* = 0.004, $\eta_{\text{p}}^{2}$ = 0.546) and condition (*F*_(2,22)_ = 1.7, *p* = 0.199, $\eta_{\text{p}}^{2}$ = 0.137). A medium-to-large effect size was present for the interaction effect (*F*_(2,22)_ = 1.5, *p* = 0.251, $\eta_{\text{p}}^{2}$ = 0.118). *Post hoc* analyses indicated that there was a small-to-medium effect on inhibitory control following the circuit condition [*M*_(SD)_ = 12.3 (15.9); *t*_(11)_ = 2.7, *p* = 0.022, *d*_rm_ = 0.37]. Further, there was a small effect on inhibitory control following the treadmill [*M*_(SD)_ = 7.3 (14.1); *t*_(11)_ = 1.8, *p* = 0.098, *d*_rm_ = 0.23] and sedentary conditions [*M*_(SD)_ = 2.8 (7.9); *t*_(11)_ = 1.2, *p* = 0.242, *d_rm_* = 0.23]. Change in inhibitory control by condition is presented in Figure 4.

### Secondary Analyses

Descriptive statistics summarizing the affect, perceived mental exertion, motivation, and task self-efficacy scores are shown, by condition, in Table 2. Results indicated that no significant differences between the conditions were present for affect, perceived mental exertion, motivation, or self-efficacy, with effect sizes ranging from small to large. Given that these analyses were secondary, non-significant effects were not further explored through *post hoc* analyses.

**Table 2:** Change in secondary outcomes by condition.

Variable	Sedentary Mean (SD)	Circuit Mean (SD)	Treadmill Mean (SD)	*F*	*p*-value	Effect size ($\eta_{\text{p}}^{2}$)
Task affect (Δ pre- to- post-test)	0.9 (0.8)	0.1 (0.6)	1.0 (1.2)	3.4	0.06	0.273
Task RPME (Δ pre- to- post-test)	−0.03 (1.6)	−0.6 (2.1)	0.7 (1.1)	1.5	0.25	0.145
Task motivation (Δ pre- to- post-test)	0.1 (0.9)	0.2 (1.2)	−0.1 (1.1)	0.26	0.77	0.026
Task self-efficacy	5.4 (2.1)	6.7 (2.9)	6.8 (3.0)	1.4	0.27	0.114

*Note: RPME, ratings of perceived mental exertion*.

### Manipulation Checks

Results from the manipulation checks are presented in Table 3 and indicate a large effect for the differences in heart rate and perceived physical exertion between the two exercise conditions and the control condition, respectively. There was also a large effect on the differences in ratings of perceived mental exertion, with participants rating the circuit condition as being the most mentally exerting. Finally, participants had the highest affect following the circuit condition, followed closely by the treadmill and sedentary conditions, respectively; this effect was medium-to-large in magnitude.

**Table 3:** Manipulation checks by condition.

Variable	Sedentary Mean (SD)	Circuit Mean (SD)	Treadmill Mean (SD)	*F*	*p*-value	Effect size ($\eta_{\text{p}}^{2}$)
Heart rate	89.7 (10.2)	131.5 (8.7)**	128.8 (10.4)**	79.0		#x0003C;0.001	0.898
Affect	2.4 (2.4)	3.5 (1.5)	3.0 (1.8)	1.5	0.24	0.133
RPE	3.2 (3.5)	6.5 (2.6)*	5.9 (3.2)*	13.5	<0.001	0.600
RPME	3.7 (3.6)	5.4 (3.3)	5.0 (3.5)	2.8	0.12	0.239

*Significantly different from the sedentary condition at p < 0.01. **Significantly different from the sedentary condition at *p* < 0.001. *Note*: RPE, Ratings of Perceived Exertion; RPME, Ratings of Perceived Mental Exertion.

## Discussion

Overall, findings from this exploratory study suggest that exercise may be a promising modality to elicit changes in cerebral oxygenation and inhibitory control in children with ASD, with effect sizes that were medium-to-large in size. While both exercise conditions led to an increase in inhibitory control, only the circuit condition led to an increase in cerebral oxygenation; thus, pointing to potentially greater benefits of interval-type exercise for improving executive functioning in children with ASD. Further, the magnitude of our effect sizes suggests that these changes are of practical significance (Ellis and Steyn, 2003; Sullivan and Feinn, 2012).

This is the first study to examine the acute effect of two different types of exercise on cerebral oxygenation and inhibitory control, when compared to a sedentary control condition, in children with ASD. Importantly, the study utilized numerous manipulation checks to further discern the effect of exercise on our primary outcomes. Results from the manipulation checks indicated that exercise intensity was similar between both exercise conditions, with no differences in the participants’ heart rate or perceived exertion; thus, it is likely that differences in our primary outcomes between the conditions may be due to mechanisms other than exercise intensity.

Previous research has demonstrated greater improvements in executive functioning following cognitively engaging physical activity when compared to traditional aerobic activity (Best, 2010; Schmidt et al., 2015; Diamond and Ling, 2018), which may be one possible reason for the differential effects between the circuit and treadmill conditions. Even though perceived mental exertion was similar between conditions, possibly the circuit condition required a greater degree of cognitive demand than the treadmill condition due to the need to switch between the different exercises and the more dynamic nature of each exercise in comparison to treadmill walking. Indeed, the exercises included in the circuit condition required a higher degree of motor coordination, given the full-body, dynamic nature of each of the five exercises. There is a high degree of co-activation between the cerebellum, responsible for motor coordination, and the prefrontal cortex, responsible for executive functions (Diamond, 2000). As such, tasks that require both motor and cognitive demands should inherently lead to greater co-activation of brain regions. It has further been proposed that the benefits of cognitively engaging physical activity can be attributed to a theory of “pre-activation” (Budde et al., 2008; Schmidt et al., 2016), whereby the cognitive engagement of the physical activity primes the same cognitive processes needed for the later performance of cognitive tasks. This theory of pre-activation is supported by the fact that we saw greater improvements in inhibitory control following the circuit exercise, which likely required a higher degree of cognitive engagement, in comparison to the treadmill exercise, which relied more on motor processes alone. Further, we saw an increase in oxy-Hb following the circuit condition, yet virtually no change in this outcome following the treadmill condition; thus, suggesting that this pre-activation effect may only be present when a certain degree of cognitive engagement occurs alongside physical activity.

Contrary to our initial hypotheses, we did not find differences between the conditions in our secondary psychological outcomes. Perhaps, the physical activity does not have the same effect on these psychological mechanisms including affect, motivation, and self-efficacy among children with ASD compared to children with typical development (e.g., Schmidt et al., 2016). However, these results may mean that children with ASD can improve their inhibitory control following exercise, regardless of their motivation or self-efficacy toward the cognitive task. This is a potentially promising finding that should be further explored in future intervention work as providing a bout of exercise before cognitive tasks, such as behavioral treatments or school activities, may be able to elicit improved outcomes on these activities, regardless of the child’s feelings toward the activity.

Although not a primary outcome, another important finding from this study was that all participants were able to complete both exercise conditions, with 100% adherence, while staying in the target heart rate zone. This finding suggests that exercise is a feasible intervention for children with ASD, which is important for the implementation and uptake of exercise-based programs for this population. Beyond replication of these study findings, an important next step in this work will also be to explore the role of exercise on executive functions outside of the lab setting. To this extent, circuit-based exercise may be a more feasible workout for school-age children with ASD who may not have access to specialized exercise equipment, such as a treadmill. While basic equipment (e.g., resistance band, medicine ball) was used for the circuit, it could also be completed without any equipment as a complete body-weight exercise session. Thus, the circuit workout could reasonably be completed in a home, school, or clinic environment with little space or equipment needed. Further, the larger effect on inhibitory control and cerebral oxygenation evident following the circuit condition suggests that this type of exercise may also elicit the greatest improvements to various health and behavioral outcomes (both acutely and overtime) in children with ASD.

Finally, while the exercise sessions were 20-min in length, it is important that future research also explores the effects of shorter bouts of activity (e.g., Howie et al., 2015; Schmitz Olin et al., 2017) to further improve the feasibility of implementing exercise breaks into multiple settings. Given that current behavioral treatment practices for this population are both time and cost-intensive (Virués-Ortega, 2010; Buescher et al., 2014), exercise may provide a relatively low-cost alternative to augment current practices. For example, the provision of a brief exercise bout either immediately before or during traditional behavioral therapy may help to improve participants’ attention and inhibition, essentially boosting the benefits of the session. Future research should build on this work by exploring the effect of exercise breaks on traditional treatment outcomes for children with ASD.

One limitation of this study is the relatively small sample that was employed. While we were limited to 12 participants due to challenges with recruitment, our *post hoc* sensitivity analysis revealed we were powered to only detect large effects. Recruitment of participants with ASD in a community setting is a challenge due to low prevalence rates and lack of interest in exercise-based studies among parents of children with ASD. Nonetheless, our sample size is not that different from previous experimental exercise-based studies in this population (Anderson-Hanley et al., 2011; Schmitz Olin et al., 2017). A second limitation is that we only included one measure of executive functioning in the study. The cancellation task we employed was considered a measure of inhibitory control; however, most studies of this aspect of executive functioning use a Flanker task to assess this outcome (Raine et al., 2018; Pontifex et al., 2019). Pilot testing in our lab suggested that the completion of a Flanker task would not be feasible for our participants. Third, our research design and measures cannot disentangle why the circuit task resulted in a larger change in cerebral oxygenation and inhibitory control in comparison to the treadmill task. It is possible that switching between exercises in the circuit required more executive functioning and greater recruitment of oxygenated hemoglobin than the treadmill. However, it is also possible that the physiological response to the interval-based workout was more beneficial than the aerobically based treadmill task. Future work should attempt to recruit larger samples, including multiple measures of executive functioning, and further, disentangle the mechanisms driving these effects.

Our study is strengthened by employing a within-subject pre-test post-test experimental design, which is considered the gold-standard for testing the acute effects of exercise on executive functioning (Pontifex et al., 2019). Another strength of this study is the inclusion of direct measures of cerebral oxygenation, inhibitory control, and psychological outcomes within the same study; something that has not previously been done in a sample of children with ASD. Further strengths are the inclusion of two different types of exercise, in comparison to a sedentary control condition, along with numerous manipulation checks.

## Conclusion

Overall, results from this exploratory study suggest that an acute bout of exercise may improve cerebral oxygenation and inhibitory control among children with ASD, with exercises that are more cognitively and motorically demanding potentially having the greatest benefits. However, further research with larger sample sizes is needed to truly establish the acute effect of exercise on executive functioning in this population.

## Data Availability Statement

The datasets generated for this study are available on request to the corresponding author.

## Ethics Statement

This study was carried out following the recommendations of Hamilton Integrated Research Ethics Board with written informed consent from all parents of participating children. All parents of participating children gave written informed consent following the Declaration of Helsinki. All child participants gave written informed assent.

## Author Contributions

EB designed the study, coordinated and carried out recruitment and data collection, conducted the data analyses, and drafted the initial manuscript. JG advised on study design and assisted in data collection. JH advised on study design and data collection. JC supervised the design and execution of all phases of the study. All co-authors reviewed and approved the final manuscript.

## Conflict of Interest

The authors declare that the research was conducted in the absence of any commercial or financial relationships that could be construed as a potential conflict of interest.
